# Death of Dr. A. Hermann

**Published:** 1874-03-15

**Authors:** 


DEATH OF DR. A. HERMANN.—On the seventh day of January, 1874, inexorable death snatched from the midst of his many friends, Dr. A. Hermann, of Pesth, the author of the series of articles on pneumonitis, now in translation in THE EXAMINER. The perusal of the abbreviated papers cannot but call forth admiration for his thorough, logical, and careful methods of research, his keen appreciation of facts, and the spirit of candor pervading his writings—of which a large variety and number have preceded his present one—and convince the reader of the great loss to science by the untimely death (at the age of thirty-seven years) of such a talented and promising disciple. The cause of his departure is stated, by some, as acute muscular rheumatism; while others speak of hydrophobia, following a slight injury from a dog's teeth, which was neglected till it ended in this fearful manner. H. G.

